# The Relationship Between Salivary Alpha Amylase Activity and Score of McGill Pain Questionnaire in Patients With Tension Type Headache

**DOI:** 10.29252/nirp.bcn.9.1.59

**Published:** 2018

**Authors:** Mohammad Vahedi, Mehrdokht Mazdeh, Mehrdad Hajilooi, Maryam Farhadian, Yasamin Barakian, Parastoo Sadr

**Affiliations:** 1. Department of Oral Medicine, School of Dentistry, Hamedan University of Medical Sciences, Hamedan, Iran.; 2. Dental Research Center, Hamedan University of Medical Sciences, Hamedan, Iran.; 3. Department of Neurology, School of Medicine, Hamedan University of Medical Sciences, Hamedan, Iran.; 4. Department of Immunology, School of Medicine, Hamedan University of Medical Sciences, Hamedan, Iran.; 5. Research Center for Health Sciences, Hamedan University of Medical Sciences, Hamedan, Iran.; 6. Department of Biostatistics, School of Public Health, Hamedan University of Medical Sciences, Hamedan, Iran.; 7. Department of Oral Medicine, Faculty of Dentistry, Qom University of Medical Science and Health Services, Qom, Iran.; 8. Department of Oral Medicine, Faculty of Dentistry, Mazandaran University of Medical Sciences, Sari, Iran.

**Keywords:** Pain measurement, Salivary Alpha Amylase, Tension-type headache

## Abstract

**Introduction:**

Tension-type headache is the most common type of headache across the world. Saliva as a non-invasive medium is used to detect a wide range of diseases. Salivary Alpha-Amylase (SAA) levels has been suggested as a potential indirect marker for detecting Sympathoadrenal Medullary (SAM) activity, which is activated by pain. Significant correlation was found between SAA levels and pain scale in patients with chronic pain. The purpose of the present study was to measure SAA activity in Frequent Episodic Tension-Type Headache (FETTH). In addition to the Visual Analogue Scale (VAS), we intend to assess intensity and various aspects of pain by McGill Pain Questionnaire (MPQ).

**Methods:**

A total of 45 females with FETTH (case group) and 45 healthy voluntary females (control group) were enrolled in our case-control study. Unstimulated saliva by spitting method was taken from each participant.

**Results:**

SAA levels were significantly higher in patients with FETTH (P<0.001) when compared with the control group. There was significant correlation between SAA activity and MPQ score (P<0.001).

**Conclusion:**

This is the first study using MPQ as a subjective means of assessing quality and quantity of pain alongside the VAS as an objective tool for evaluating pain in patients with FETTH. SAA may be an appropriate marker for assessing of pain levels in patients with FETTH. MPQ versus the VAS may be a more accurate measurement tools along VAS.

## 1. Introduction

Tension-Type Headache (TTH) is the most common type of headache in all age groups across the world. Because of its high prevalence, it has the greatest social and economic impact in comparison with other primary headaches (Crystal & Robbins, 2010). Saliva as a non-invasive medium is used to detect a wide range of diseases (Rantonen, 2003). Alpha-amylase makes up 40%–50% of total salivary proteins. Release of Salivary Alpha Amylase (SAA) increases during psychosocial stress (Nater & Rohleder, 2009). Measuring SAA levels as a potential indirect marker for assessing of Sympatho-adrenal Medullary (SAM) activity has been suggested by many researchers (Bugdayci, Yildiz, Altunrende, Yildiz, & Alkoy, 2010; Rohleder, Wolf, Maldonado, & Kirschbaum, 2006; Van Stegeren, Rohleder, Everaerd, & Wolf, 2006). Pain activates SAM and hypothalamic-pituitary-adrenal axis. Significant correlation was found between SAA levels and pain scale in patients with chronic pain (Shirasaki et al., 2007). TTH is divided into four types according to the latest classification of International Headache Society (IHS); infrequent episodic, Frequent Episodic (FE), chronic type, and probable tension-type headaches (Headache Classification committee of the International Headache Society, 2013). The prevalence of episodic form is 38% in adults and decreases with rising age (Crystal & Robbins, 2010).

For pain assessment and treatment response, pain scales can be helpful (Tandon et al., 2016). These scales can measure intensity and other features of pain. The measures are classified into three groups that included self-report, observational (behavioral), and physiological data. The Visual Analogue Scale (VAS) is a sensitive subjective scale in expressing pain severity (Tripathi & Kumar, 2014). On the other hand, McGill Pain Questionnaire (MPQ) is a comprehensive multidimensional tool that encompasses neurophysiological and psychological scopes of pain (Ngamkham et al., 2012). In addition to pain intensity, quality of pain experience (sensory, affective, evaluative, and other dimensions of pain) is assessed by MPQ (Burckhardt & Jones, 2003; Holroyd et al., 1992; Melzack, 1975). MPQ can provide more detailed and clinically information about pain experience of patients with headache (Majani et al., 2003). There was a significant correlation between VAS pain scale and SAA. SAA is suggested as a good index for the objective assessment of pain intensity (Shirasaki et al., 2007).

The purpose of the present study was measurement of SAA activity in TTH. In addition to VAS, we measured intensity and various aspects of pain (sensory, affective, evaluative, and miscellaneous) by MPQ to show any association between SAA and subjective scales mentioned.

## 2. Methods

This study was a case-control investigation. A total of 45 females with FETTH (case group) and 45 healthy voluntary females (control group) were enrolled in the present study. Informed consent was obtained from all subjects. All protocols and patient informed consent forms were approved by the Ethics Committee of Hamadan University of Medical Sciences (Approval number: p/16/35/9/4681). All participants were aged between 25 and 45 years. Inclusion criteria include having IHS criteria confirmed by neurologist and also the absence of headache during sample collection. Exclusion criteria included having chronic disease (cardiovascular, endocrine, psychiatric, etc.), history of drug treatment (beta-blockers, psychoactive drugs, or glucocorticoids), current cigarette smoking, alcohol consumption, pregnancy, eating, drinking, or consuming any oral stimulation 90 minutes prior to sample collection.

Necessary to declare that all protocols and patient informed consent forms were approved by the Ethics Committee of Hamadan University of Medical Sciences (Approval number: p/16/35/9/4681).

### 2.1. Collection of saliva

After explaining the process and before saliva sampling, demographic information form for both groups, Persian version of MPQ (Khosravi, Sadighi, Moradi, & Zendehdel, 2013), and VAS in case group were completed via interview. Then 2–5 mL of unstimulated saliva in 5 minutes was taken from each participant in a sterile plastic container by spitting method (Tandon et al., 2016). Participants were in a sitting position while bending the neck forward. To exclude the possible effects of diurnal rhythm, collection of saliva was performed between 4–6 PM. Saliva sampling was stored at −20°C until laboratory analysis (Nater & Rohleder, 2009).

### 2.2. Sialochemical analysis

After defrosting on ice, the saliva samples were centrifuged (Sigma 112 USA) for 3–5 minutes to remove any particles. Then saliva was diluted (1:100) with distilled water. The level of SAA activity was assessed by biochemical kit (EPS-G7, Pars Azmoon Co., Karaj, Iran) (Ahmadi-Motamayel, et al., 2016; Ahmadi-Motamayel et al., 2013; Faulkner & Meites, 1982) and a spectro-photometer (BS-380 Mindray, China) at a wavelength of 405 nm. In this method, the reaction of alpha-amylase on a chromogenic substrate produces a colored solution of chloro-p-nitrophenol and its darkness is proportional to the level of enzyme activity.

### 2.3. Statistical analysis

The obtained data were analyzed by SPSS 21. In order to compare two groups, t test and Mann-Whitney nonparametric test were used respectively for evaluating variables with normal distribution (age) and variables with nonparametric distribution (Body Mass Index [BMI] and SAA activity). Pearson correlation coefficient was used for the assessment of the relationship between SAA activity with age, aspects of sensory, affective, miscellaneous, and total score of MPQ. To investigate the association between SAA activity with VAS, BMI and evaluative aspect of MPQ, the Spearman correlation coefficient was used. To assess the relationship between VAS and MPQ, Pearson correlation coefficient was used. In all analyses, the significance level was set as P<0.05. Multiple linear regressions were applied to detect the relationships between SAA activity with age, BMI, and MPQ score in case group.

## 3. Results

**Figure 1.**
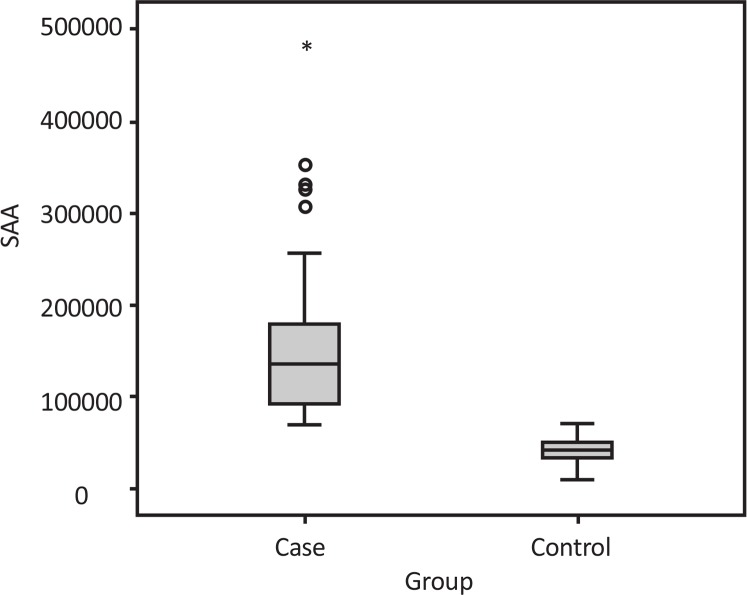
Salivary alpha-amylase activity in case and control group

The mean (SD) SAA activity was 160295.55(87951.12) unit/L (range 69000–479000) in the case group and 42135.55(14635.65) unit/L (11600–71200) in the control group (Figure 1). Descriptive analyses of age, BMI, and SAA activity levels are presented in Table 1. There was no statistical difference with regard to age (P=0.455) and BMI (P=0.626) between the control and case groups. Significant difference was found between the control and case groups with regard to SAA activity levels (P<0.001). The mean (SD) of MPQ total score (MPQTS) was 30.97(6.82) (range 20–47).

**Table 1.** The mean and Standard Deviation (SD) of the age, BMI, and the level of SAA activity of the study participants

	**Number**	**Age, y**	**BMI, kg/m^2^**	**SAA Activity (Unit/L)**

		**Mean±SD**		
Case	45	34.37±6.65	23.71±1.17	160295.55±87951.12
Control	45	33.40±5.65	23.55±1.30	42135.55±14635.65
P value		0.455	0.626	0.000000

**Figure 2.**
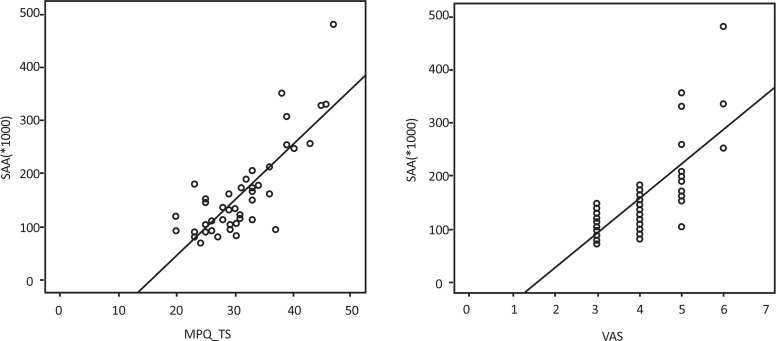
Scatter plot of SAA activity, MPQ_TS (2A) and VAS (2B) in individuals with frequent episodic tension type headache

The results of the correlation between salivary amylase and variables in the case group are summarized in Table 2. Significant correlation was between SAA activity and MPQ score (r=0.813, P<0.001) (Figure 2A). Significant correlation was observed between SAA activity and VAS score (r=0.743, P=0.000) too (Figure 2B). The Pearson correlation analysis showed significant statistical correlation between VAS and MPQ score (P<0.001). The Pearson correlation analysis didn‘t show significant statistical correlation between age and SAA activity in two groups (P=0.078). Moreover, there was no significant correlation between SAA activity and BMI in two groups (P=0.431). To investigate the association of SAA with age, BMI and total score of MPQ, linear regression model was used. Multiple linear regression showed positive correlation between SAA activity and MPQ score (Beta=9178.69, P<0.001).

**Table 2.** The correlation coefficient between SAA activity and variables in case group

	**Age**	**BMI**	**VAS**	**Sensory**	**Affective**	**Evaluative**	**Miscellaneous**	**MPQ-TS**
Correlation coefficient	0.266	0.120	0.743	0.594	0.457	0.278	0.648	0.813
P	0.078	0.431	0.000000	0.000017	0.002	0.065	0.000002	0.000000

## 4. Discussion

In the present study, there was statistically significant difference between two groups so that the mean SAA levels were higher in the case group. Also In the patient group, the higher SAA level was proportional to MPQ score and VAS score.

Salivary biomarkers such as amylase have been known as stress biomarkers. Since a psychological stress and painful stimuli could activate SAM system, SAA may work as an easy index for assessing of SAM activity and indirect marker for the objective assessment of pain (Bugdayci et al., 2010; Shirasaki et al., 2007; Tsuchiya, Saidin, Inoue, Kajiwara, & Kimura, 2014).

In the literature, we found no study about the SAA activity levels in the patients with TTH. In one study on migraine headache (Bugdayci et al., 2010), SAA levels diminished during the attack period in moderate and severe pain that is inconsistent with the present study. Since stress is considered as a contributing factor in TTH (Cathcart, Winefield, Lushington, & Rolan, 2010), this discrepancy may be due to a greater role of stress in TTH. In another study on patients with chronic pain, a significant positive correlation was reported between SAA levels and pain scale (Shirasaki et al., 2007). In another study, the level of SAA was correlated with the pain severity in patients with symptomatic irreversible pulpitis (Ahmadi-Motamayel et al., 2013). The results of two recent studies were similar to the present study. In the present study, SAA activity showed a significant correlation with VAS, more over SAA was sufficient for objective assessment of the pain experimented by patients with FETTH. This finding is consistent with studies results of Ahmadi-Motamayel et al. (2013), Shirasaki et al. (2007), and Noto, Sato, Kudo, Kurata, & Hirota, (2005).

In addition to VAS, we used MPQ for the evaluation of quality and quantity of pain. MPQ comprised 78 descriptive words in 20 subgroups. This questionnaire evaluates aspects of pain, including sensory (1:11), affective (11–15), evaluative (16) and miscellaneous (subgroup 17–20) (Khosravi, Sadighi, Moradi, & Zendehdel, 2013).

A meta-analysis concludes that normative scores of MPQ across painful conditions range from 24% to 50% of the maximum scores (Burckhardt & Jones, 2003; Wilkie, Savedra, Holzemer, Tesler, & Paul, 1990). In this study, mean MPQ score was 30.977. The same study was not available to compare the different aspects of MPQ. The results of this study showed that total score of MPQ increased along with the level of SAA activity. We didn’t find similar studies conducted on SAA accompanied with MPQ.

Given that the correlation between SAA activity and MPQ score is more than the correlation between SAA activity and VAS, we may conclude that MPQ is a more accurate subjective tool than VAS to reflect increased SAA (objective measurement tool) in TTH. Majany and colleagues showed that in the field of headache and cancer, MPQ can provide more detailed and clinically useful information about patients’ pain experiences (Majani et al., 2003).

In the present study, among all aspects of the MPQ, the highest correlation was observed between SAA level activity and miscellaneous aspects of MPQ. This aspect is composed of four questions that its first two questions have the most propinquity with clinical characteristics of TTH. According to this result, perhaps we can conclude that increased SAA levels represent better pain features such as the distribution of pain, severity of tightness, feeling of coldness, and a nettlesome severity of pain in FETTH. Future studies are mandatory to examine SAA activity in other primary headaches, especially before and after treatment.

According to the result of the present study, SAA activity in patients with FETTH has increased compared with the control group. Also there was significant correlation between SAA activity and MPQ score. So SAA may be an appropriate marker for assessing of pain levels in patients with FETTH, perhaps we can conclude that MPQ is a more accurate subjective tool than VAS to reflect increased SAA (as an objective measurement tool) level in FETTH.
